# P07 The use of JAK inhibitors in difficult-to-treat adult-onset Still’s disease

**DOI:** 10.1093/rap/rkae117.038

**Published:** 2024-11-05

**Authors:** Eman Elfar, Arti Mahto

**Affiliations:** King’s College Hospital, London, United Kingdom; King’s College Hospital, London, United Kingdom

## Abstract

**Introduction:**

Adult-onset Still’s Disease (AOSD) is a rare systemic auto-inflammatory disorder characterised by fever, arthritis and rash. Some patients with AOSD exhibit resistance or intolerance to conventional treatments, necessitating alternative therapeutic strategies. This case explores the efficacy and safety of janus kinase (JAK) inhibitors in managing difficult-to-treat AOSD. The driving force behind Still’s disease lies in the excessive and inappropriate production of pro-inflammatory cytokines, notably interleukin-6 (IL-6) and interferon (IFN). Given the pivotal role of JAK inhibitors in mitigating these cytokine pathways, this study highlights their potential therapeutic impact in the management of difficult-to-treat AOSD.

**Case description:**

A 32-year-old male presented in July 2021 with a three-week history of fevers, night sweats, rigors, and weight loss. A CT scan revealed widespread lymphadenopathy above and below the diaphragm. Blood cultures were negative and, despite antibiotics, the patient continued to experience intermittent fevers. Anemia was asymptomatic, and the patient was transfused with two units of blood. Chronic B infection led to the initiation of entecavir. The patient also experienced palpitations and tachycardia, with an echo revealing a small pericardial effusion, hypertrophied and hyperdynamic left ventricle (LV) with an ejection fraction of 70-75%. A lymph node biopsy was inconclusive, prompting a second biopsy.

In September 2021, hyponatremia due to SIADH developed. The patient had ongoing symptoms, including weight loss, and a bone marrow biopsy showed reactive features. CT and PET scans revealed generalised lymphadenopathy. Rheumatology consultation in November 2021 indicated intermittent fever, weight loss, joint pain, and elevated inflammatory markers. The patient had dermatographic lymphadenopathy, and a negative lymph node biopsy lowered the probability of HLH. Methotrexate was initiated, later switched to azathioprine and prednisolone due to ongoing symptoms.

In June 2022, the patient started tocilizumab, which resolved joint pain but caused ongoing dermatitis. Tocilizumab was discontinued in January 2023 due to inefficacy, and anakinra was initiated. Prednisolone was gradually tapered off. The patient experienced shortness of breath, and echo revealed pulmonary hypertension. Tocilizumab was discontinued due to injection site reactions and increased hepatitis B virus titers. In March 2023, the decision was made to start the JAK inhibitor tofacitinib. By May 2023, ferritin levels were 1196, and the patient was successfully coming off prednisolone with well-controlled symptoms.

**Discussion:**

This case underscores the efficacy of JAK inhibitors as a successful therapeutic intervention in challenging cases of AOSD. The 32-year-old male presented with severe symptoms including fevers, night sweats, weight loss, and widespread lymphadenopathy. Initial treatments with antibiotics, methotrexate, azathioprine, prednisolone, and tocilizumab were either ineffective or led to adverse reactions.

Despite these setbacks, the patient showed a remarkable clinical response to the JAK inhibitor tofacitinib, initiated in March 2023. The swift and sustained improvement in symptoms, along with a favourable safety profile, suggests that JAK inhibitors can be a promising treatment for refractory AOSD. The ability to taper off steroids while maintaining symptom control is particularly noteworthy, as long-term steroid use is associated with significant side effects.

The success of JAK inhibitors in this case is likely attributed to their mechanism of action, which strategically blocks pro-inflammatory cytokine pathways, particularly IL-6 and IFN. This targeted approach addresses the underlying pathophysiology of AOSD, leading to better disease management.

However, this case also highlights the importance of monitoring for potential side effects, such as increased hepatitis B virus titres observed in this patient. This necessitated adjustments in therapy and careful monitoring of liver function.

The encouraging outcomes in this case prompt the need for further prospective studies to validate these findings. Future research should focus on elucidating the optimal dosing and duration of JAK inhibitor therapy in patients with refractory AOSD. Additionally, long-term follow-up studies are essential to fully understand the safety profile of these agents.

**Key learning points:**

• **Refractory AOSD treatment**: JAK inhibitors, such as tofacitinib, can be effective for patients with refractory AOSD who do not respond to conventional therapies.

• **Steroid sparing**: JAK inhibitors may allow for the tapering off of steroids, reducing the risk of long-term steroid-related side effects.

• **Cytokine pathway targeting**: The success of JAK inhibitors highlights the importance of targeting specific pro-inflammatory cytokine pathways, particularly IL-6 and IFN, in managing AOSD.

• **Monitoring side effects**: Continuous monitoring for potential side effects, including increased the risk of thromboembolic events is crucial when using JAK inhibitors.

• **Need for further research**: Prospective studies are needed to validate the efficacy and safety of JAK inhibitors in AOSD and to determine optimal dosing and treatment duration.

